# Secular trends in age at menarche among women born between 1955 and 1985 in Southeastern China

**DOI:** 10.1186/s12905-014-0155-0

**Published:** 2014-12-12

**Authors:** Yanyu Lyu, Lucia Mirea, Junmin Yang, Ruth Warre, Jun Zhang, Shoo K Lee, Zhu Li

**Affiliations:** Department of Child Health Development, Capital Institute of Pediatrics, Beijing, China; School of Public Health, Peking University Health Science Center, Beijing, China; Maternal-Infant Care Research Centre, Mount Sinai Hospital, Toronto, Ontario Canada; Dalla Lana School of Public Health, University of Toronto, Toronto, Ontario Canada; MOE-Shanghai Key Laboratory of Children’s Environmental Health, Xinhua Hospital, School of Medicine, Shanghai Jiao Tong University, Shanghai, China; Department of Paediatrics, University of Toronto, Toronto, Ontario Canada

**Keywords:** Epidemiology, Puberty, Menarcheal age, Socio-economic status, China

## Abstract

**Background:**

Improvements in socioeconomic conditions and population health have been linked to declining age at menarche. In China, secular trends in age at menarche following extensive economic reform during recent decades have not been thoroughly investigated. This study examined the overall trend in age at menarche and assessed differences in the rate of change of age at menarche over time, and between urban and rural populations and education levels in southeastern China.

**Methods:**

Age at menarche was retrospectively collected from 1,167,119 Han Chinese women born 1955–1985, who registered in the Perinatal Health Care Surveillance System in 19 cities and counties in two southeast provinces during 1993–2005. Multivariable linear regression was used to estimate trends in age at menarche overall and stratified by urban/rural residence and education level.

**Results:**

Age at menarche declined by 0.33 [95% CI 0.33, 0.32] years/decade overall, with the fastest decline in women born in 1966–1975. For the earliest birth cohorts (1955–1965), age at menarche declined faster in urban versus rural regions, and for women with high school education or above versus primary school or less. In contrast, age at menarche declined slower among urban women born 1976–1985, and among those with higher education born 1966–1985.

**Conclusions:**

Mean age at menarche declined for women born in 1955–1985 in southeast China. Further study is warranted to identify specific factors contributing to earlier age at menarche and associated health outcomes.

**Electronic supplementary material:**

The online version of this article (doi:10.1186/s12905-014-0155-0) contains supplementary material, which is available to authorized users.

## Background

Age at menarche (AAM) is an important event in a woman’s life and is affected by both biological and environmental factors, such as genetics, body weight, nutrition, and socioeconomic status [1,2]. Earlier AAM can negatively impact social and behavioral development [3,4], and may increase risk of long-term health outcomes including obesity, breast cancer, type 2 diabetes, and mortality [5-8]. In addition, reports indicate that earlier AAM in mothers can increase their offspring’s risk of obesity [9]. Multiple sources of evidence indicate that AAM declined in Europe and the United States between the 1830s and 1970s, with rates of decline ranging from 3.2 months/decade in Scandinavian countries to 1.1 month/decade in France [10]. One hypothesis suggests that AAM decreased due to the vast improvement in socioeconomic conditions, including higher gross domestic product (GDP), better nutrition, reduced infectious diseases, and overall better health outcomes [4,11] in western countries during that time period. Studies examining AAM among women born after 1950 in western countries have provided conflicting results, with reports indicating a continued but slower decrease in AAM [12], constant AAM [13-15], or increasing AAM [16,17].

Among developing countries, inequalities related to environment and lifestyle (urban vs. rural), as well as socioeconomic status persist and likely contribute to AAM. Indeed, variations in secular trends of AAM have been observed in developing countries, likely due to differing changes in living standards [18]. For example, a study from South America indicated that younger AAM, previously more common among girls from affluent backgrounds, has become more common among girls with lower-socioeconomic status and that AAM is also declining more rapidly in these girls compared with girls from affluent backgrounds [19]. In the past four decades, China has undergone great economic reform that has improved living conditions in many regions in a relatively short period of time. Decreasing AAM in China has been reported in retrospective studies including those examining participants in the Chinese Two-per-Thousand Fertility Survey born between 1950 and 1973 [20] and women included in the China Kadoorie Bank born between 1930 and 1974 [21], with earlier AAM more pronounced among women living in urban regions. More recent cross-sectional surveys of Chinese girls aged 8 to 18 years, including the Chinese National Survey on Students’ Constitution and Health conducted in 1985, 1995, 2000, 2005, and 2010 [22-24], and the National Physical Fitness and Health Surveillance conducted in 1995 and 2005 [25] reported continued AAM decline; however, rates of decline were faster in rural compared with urban regions. No previous population-based study has estimated the rate of change in AAM for birth cohorts spanning the time period in which the greatest improvements in living conditions and health were achieved in China.

This study examined secular trends in AAM for women born in 1955 to 1985 in southeast China using population-based data from the Perinatal Health Care Surveillance System. Our specific objectives were to: 1) estimate the overall trend in AAM and characterize differences in the rate of AAM change across time; and 2) assess differences in the rate of AAM change between urban and rural populations and education levels.

## Methods

### Study population, ethics, and data collection

This retrospective study examined data from women who registered in the Perinatal Health Care Surveillance System (PHCSS) between 1993 and 2005. The PHCSS was established by the National Center of Maternal and Infant Health (NCMIH) at Peking University Health Science Center in 1993 as an essential component of the population-based China-U.S. Collaborative Project for Neural Tube Defect Prevention, with population coverage of approximately 20 million from 27 cities and counties in China (6 in one northern province and 21 in two southeastern provinces). Details describing the PHCSS have been published previously [26,27]. Data collection and all methods used in the surveillance program were approved by the institutional review board of Peking University Health Science Center. Oral informed consent was obtained from every woman as per the PHCSS protocol [28,29]. Women residing in regions covered by the PHCSS registered at their marital physical examination or first prenatal visit. Data collected at the time of registration and at follow-up visits included demographics, medical and reproductive history, prenatal care provided, complications, delivery summary, and postpartum conditions. Data were entered into the PHCSS database electronically by trained staff at the local sites. De-identified registry data collected at the surveillance sites were sent to NCMIH for data cleaning and inclusion into the PHCSS. Missing and erroneous data were sent back to hospitals for data correction.

Audit of data quality and completeness during the design of this study indicated that electronic records for the northern province were not complete after 2000, possibly due to the introduction of an advanced electronic hospital record system that occurred only in the two southeastern provinces in 2001. Therefore, to avoid selection bias, study subjects were restricted to Han Chinese women born between 1955 and 1985 from 19 cities and counties in the two southeast provinces (Zhejiang and Jiangsu) that collected data for the PHCSS without interruption from 1993 to 2005. Women with hyperthyroidism, mental health problems (manic disorder, depression, schizophrenia, etc.), and those who did not recall their AAM were excluded from the study. In addition, women who reported their AAM as more than 20 years old were excluded due to likely inaccurate recall or possible hormone abnormalities [30].

### Study variables

Age at registration in the PHCSS and AAM were reported as one full year of life. Region of residence was classified as urban or rural according to Chinese administrative compartmentalization. Education levels were defined as primary school or less, middle school, high school or above; typically in state-financed educational systems, primary school is 6 years, middle school is 3 years, and high school is 3 years [31]. Data on medical history included heart disease, tuberculosis, kidney disease, liver disease, hypertension, diabetes, anemia, hyperthyroidism, and mental health problems. A health status indicator was derived using medical history data to distinguish women free of any chronic diseases from those with at least one chronic disease.

### Statistical analysis

Characteristics of the study population were summarized across 5-year birth cohorts, and differences were tested using the ANOVA F-test for continuous variables, and the Pearson χ^2^ test for categorical variables. Trends were assessed using the Cochrane-Armitage trend test and the Mantel-Haenszel test for binary and multi-level factors, respectively. Linear regression was used to examine trends for AAM and age at registration.

Within each 5-year birth cohort, mean AAM was compared between urban/rural regions using the Student’s *t*-test, and between education levels using ANOVA. Multivariable linear regression was applied to estimate the overall annual rate of change in AAM, and to test for interactions between year of birth with region and education. Subsequent analyses were stratified by region and education, and performed separately within each of three decade cohorts (1955–1965, 1966–1975, and 1976–1985). All statistical analyses were performed using SAS version 9.2 (SAS Institute Inc., Cary, NC), and all tests were two-sided and evaluated at a significance level of 0.05.

## Results

From January 1, 1993, to December 31, 2005, a total of 1 173 936 Han Chinese women registered in the PHCSS from 19 cities and counties within the two southeast provinces. The following women were excluded: 2 248 with missing information on AAM, 1 668 with missing birth year, 299 diagnosed with mental health problems, 887 with hyperthyroidism, 652 who reported their age at menarche as >20 years, and 1 063 women born before 1955 or after 1985. The final study population included 1 167 119 women of child-bearing age, of whom 163 879 (14%) resided in urban regions and 1 003 240 (86%) in rural regions.

Mean AAM declined significantly from 15.7 years for women born in 1955–1960 to 14.7 years for women born in 1981–1985 (Table 1). Age at registration ranged from 14 to 49 years with a significant decreasing trend across the 5-year birth cohorts. A significant increasing trend toward urban residence was detected, despite some variability in the estimated percent of women residing in urban/rural regions across the 5-year birth cohorts. Additional trends indicated women increasingly achieving a higher level of education, and being free of any chronic disease (Table 1).

**Table 1:** **Characteristics of women born in southeastern China between 1955 and 1985 by 5-year birth cohort**

	**1955–60**	**1961–65**	**1966–70**	**1971–75**	**1976–80**	**1981–85**	**Association**	**Trend**
	**(n = 7 128)**	**(n = 53 245)**	**(n = 309 379)**	**(n = 369 971)**	**(n = 348 356)**	**(n = 79 040)**	***P*** **Value**	***P*** **Value**
**Age at menarche (yrs), mean (SD)**	15.7 (1.5)	15.5 (1.39)	15.1 (1.3)	14.9 (1.3)	14.8 (1.2)	14.7 (1.2)	<0.0001^*^	<0.0001^†^
**Age at registration (yrs), mean (SD)**	36.2 (2.6)	32.3 (2.25)	27.3 (3.3)	25.1 (2.6)	24.4 (2.0)	22.3 (1.1)	<0.0001^*^	<0.0001^†^
**Region, n (%)**							<0.0001^‡^	<0.0001^¶^
Urban	847 (11.9)	3 902 (7.3)	39 698 (12.8)	51 565 (13.9)	58 740 (16.9)	9 127 (11.6)		
Rural	6 281 (88.1)	49 343 (92.7)	269 681 (87.2)	318 406 (86.1)	289 616 (83.1)	69 913 (88.5)		
**Education, n (%)**							<0.0001^‡^	<0.0001^§^
High school or above	876 (12.3)	6 195 (11.67)	52 373 (17.0)	78 738 (21.3)	124 960 (35.9)	25 420 (32.3)		
Middle school	2 151 (30.3)	25 278 (47.60)	167 684 (54.4)	232 677 (63.0)	200 758 (57.7)	49 501 (62.8)		
Primary school or less	4 076 (57.4)	21 634 (40.5)	88 225 (28.6)	57 768 (15.7)	22 152 (6.4)	3 897 (4.9)		
**Medical health history, n (%)**							<0.0001^‡^	<0.0001^¶^
No chronic diseases	6 854 (96.2)	51 096 (96.0)	298 418 (96.5)	360 774 (97.5)	343 415 (98.6)	78 453 (99.3)		
At least one chronic disease	274 (3.8)	2 149 (4.0)	10 961 (3.5)	9 197 (2.5)	4 941 (1.4)	587 (0.7)		

Missing age at registration, 20; Missing education information, 2 756.

^*^P value from ANOVA.

^†^P value from linear regression.

^‡^P value from Pearson Chi-square test.

^¶^P value from Cochrane-Armitage Trend test.

^§^P value from Mantel-Haenszel test.

**Figure 1:**
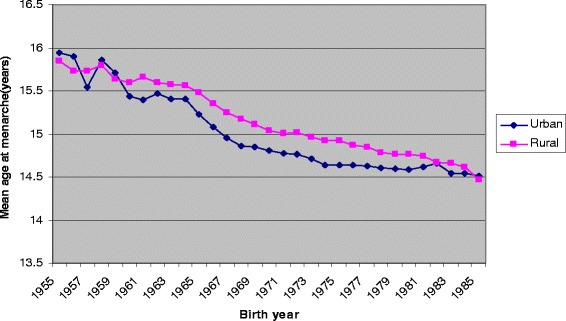
**Mean age at menarche in urban and rural women born in Southeast China between 1955 and 1985 and registered in the Perinatal Health Care Surveillance System.**

**Figure 2:**
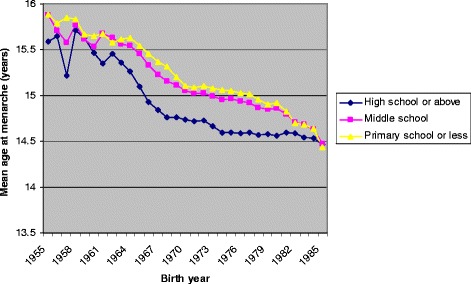
**Mean age at menarche by education level among women born in Southeast China between 1955 and 1985 and registered in the Perinatal Health Care Surveillance System.**

Among all study women, mean AAM decreased by 0.42 years/decade [95% CI 0.42, 0.41] before adjustment and 0.33 years/decade [95% CI 0.33, 0.32] after adjustment for region, education level, and age at registration. Significant interactions were detected between region and birth year (*P* < 0.0001), and between education and birth year (*P* < 0.0001). Figures 1 and 2 show the mean AAM for each birth year by region and education level, respectively. Significant decline in yearly mean AAM was detected for women from both urban and rural regions over the entire study period (Table 2, Figure 1). However, the decline in AAM was faster in urban compared with rural regions for women born in the earliest cohorts, 1955–1965. In contrast, for women born in the latest cohorts 1976–1985, AAM declined at a faster rate among those from rural compared with urban regions (Table 2). Analyses stratified by level of education detected significant yearly decline in mean AAM within all three education levels (Table 2, Figure 2). AAM declined faster among women with the lowest level of education (primary school or less) born during both 1966–1975 and 1976–1985 (Table 2).

**Table 2:** **Yearly changes in age at menarche overall and in each 10-year birth cohort by region and education for women born in southeastern China between 1955 and 1985**

**Variables**	**Overall**	**1955–65**	**1966–75**	**1976–85**
	**Coefficient [95% CI]**	**Coefficient [95% CI]**	**Coefficient [95% CI]**	**Coefficient [95% CI]**
**All** ^*^	−0.033 [−0.033, −0.032]	−0.038 [−0.046, −0.030]	−0.043 [−0.045, −0.042]	−0.032 [−0.034, −0.031]
**Region** ^**†**^				
**Urban**	−0.027 [−0.029, −0.026]^‡^	−0.044 [−0.068, −0.019]^‡^	−0.040 [−0.044, −0.036]	−0.028 [−0.033, −0.023]^‡^
**Rural**	−0.034 [−0.034, −0.033]^‡^	−0.038 [−0.046, −0.029]^‡^	−0.044 [−0.046, −0.043]	−0.033 [−0.035, −0.032]^‡^
**Education** ^**¶**^				
**High school or above**	−0.031 [−0.032, −0.030]^§^	−0.061[−0.084, −0.037]	−0.036 [−0.040, −0.033]^§^	−0.027 [−0.030, −0.023]^§^
**Middle school**	−0.033 [−0.034, −0.032]^§^	−0.040 [−0.052, −0.028]	−0.043 [−0.045, −0.041]^§^	−0.035 [−0.037, −0.032]^§^
**Primary school or less**	−0.040 [−0.042, −0.038]^§^	−0.030 [−0.042, −0.019]	−0.050 [−0.054, −0.047]^§^	−0.047 [−0.054, −0.040]^§^

^*^Adjusted for region, education and age at registration.

^**†**^Adjusted for education and age at registration.

^‡^Significant *P* < 0.05 interactions detected between region and birth year.

^**¶**^Adjusted for region and age at registration.

^§^Significant *P* < 0.05 interactions detected between education and birth year.

Among every region and education subgroup, the rate of decline in AAM was faster for women born 1966–1975 than 1976–1985 (Table 2), suggesting that the overall rate of AAM decline was slowing. However, despite variable rates of AAM decline among region and education levels, the mean AAM remained lower among urban compared with rural women, and among women with higher education level (*P* < 0.0001) within each 5-year birth cohort (Additional file 1: Table A1).

## Discussion

In this population-based study, AAM decreased by 0.33 years/decade among women born during 1955–1985 in southeast China after adjustment for urban/rural region, education level, and age at registration. The rate of AAM decline varied between urban and rural regions, education levels, and across decades of the study period.

Over the past 150 years, the occurrence of menarche has been lowered globally by 3 years, corresponding to 0.2 year/decade [32]. The observed AAM decline in our study of women from China is consistent with results from European countries, which indicate an overall decline in AMM of approximately 0.30 years/decade from the mid-19th century to the end of the 20th century [33]. In the first half of the 20th century rates of AAM decline ranged from 0.08 to 0.33 years/decade among 286,205 women from nine European countries born in 1935–1964 [34]. For American women born in 1910–1949, Nichols et al. [16] reported a decline in AAM of 0.13 years/decade. In contrast, a constant AAM of 12.8 years has been observed in American Caucasian women born after 1940 [15,35], and a constant mean AAM of 12.3 years was reported among Greek girls aged from 14.8 to 18.2 years old surveyed in 1996 and 2006 [13]. Increases in AAM have also been reported for American women born in 1960–1969 compared with those born in 1940–1949 [16], and among British university students born in 1952–1967 [17], indicating that the decline in AAM in developed countries has slowed to a stop or even reversed.

Heterogeneity of AAM trends has been attributed to social class and economic conditions including income, urban or rural region, education level, and family size, which reflect general health and nutrition [33]. In this Chinese study population, a significant, decreasing trend in AAM persisted, even after adjustment for rural/urban region, education level, and age at registration in the PHCSS, suggesting that other factors beyond these impact declining AAM. Important factors that may contribute to the observed decline in AAM in China include quantity and quality of nutrition, childhood body mass index, physical labor, sanitary standards, general health status, decreased family size due to birth control availability, and pesticide exposure before AAM [36,37]. The decline in AAM among Chinese women as observed in this study and previously [21,23-25,38,39] indicates substantial changes in factors impacting AAM in China.

The observed decline in AAM was fastest for women born in 1966–1975 who experienced puberty during the extensive economic reform in China that started in 1978. For women born in 1976–1985 and growing up following economic reform, AAM continued to decline, but at a slower rate. Improvements in nutrition are often cited as the main factor impacting AAM [40]. For example, mean AAM increased in French, German, and Dutch women who reached puberty during the Second World War; subsequently a steady decline in the mean AAM was observed [34]. However, Herman-Giddens suggested that factors contributing to the continued decline of AAM in the US changed around the 1960s “from positive ones, such as better nutrition and less infectious disease to negative ones, such as ‘overnutrition’, decline of physical activity, and increased chemical pollution” [4]. An alternative view of the factors that impact AAM is proposed by the “Developmental Origin of Health and Diseases” hypothesis. According to the hypothesis, if conditions during the fetal period are poor in nutrients or oxygen, a girl is epigenetically programmed for survival over a short life span. As such, AAM is accelerated so that birth of the next generation can occur as early as possible and ensure survival of the species. Given that the acceleration in AAM is programmed during the intrauterine period, it will happen even if the living conditions after birth are better than those of the intrauterine period. Such a mismatch between the two ‘critical windows’ of the intrauterine period and childhood (i.e., poor nutritional conditions during the fetal period followed by sufficient nutritional conditions during childhood) is also thought to increase the risk of chronic diseases, such as type 2 diabetes and cardiovascular disease, thereby shortening lifespan and producing additional epigenetic programming in early childhood to further decrease AAM [41].

In this study, the decline in AAM in China over a 30-year time span did not follow a consistent pattern, which can be explained by considering the Developmental Origin of Health and Diseases hypothesis. Women who were born in the years 1955–1965 received an insufficient amount of nutrients *in utero*, especially during the national famine from the latter half of 1959 to 1960. The process of intrauterine epigenetic programming adjusted the developing fetuses to the situation of deficiency during the fetal period and early childhood life. The Cultural Revolution of 1966–1976 had a traumatic effect on every aspect of life in China and living conditions were not greatly improved during this period. Therefore, before the onset of puberty, girls born in 1955–1965 didn’t experience any great improvements in nutrition. As such, although there was a decline in AAM in this decade, the rate of decline was slow. For women born in 1966–1975, their nutrition in the intrauterine and early childhood periods was poor. However, conditions radically improved with respect to access to food after 1978, resulting in the phenomenon of mismatch and the greatest acceleration of menarche. The slowest pace of acceleration of menarche among women born in 1976–1985 can be explained by the fact that the phenomenon of mismatch did not occur after birth and women were epigenetically programmed for conditions of adequate nutrition. Further study of the national famine period in China is needed to evaluate the critical developmental window that determines age at menarche; is it the fetal, neonatal, preschool, or school period?

Variable rates of decline in AAM were observed among urban and rural regions, and education levels over time. Among the oldest cohorts, born 1955–1965, AAM declined at a faster rate among urban compared with rural women indicating greater improvement in living conditions in urban regions. However, the rate of AAM decline was steeper among rural compared with urban women born 1976–1985 following economic reform. This is consistent with previous studies of women born during the same time period in China [22,23,39], and likely due to rural industrialization since the economic reforms initiated in 1978. These reforms included profit incentives to rural collective enterprises and allowing farmers to retain surplus produce for themselves after sale of a contracted amount to the state, which yielded unprecedented agricultural GDP growth of 12% annually from 1978 to 2007. Inverse correlations between GDP and AAM have been reported by several studies [11,42,43]. Whether the pattern of declining AAM between urban and rural women in our study relates directly to factors of economic development over the time period needs further investigation.

Rates of AAM decline varied among education levels over time with a steeper rate of decline among women born in 1966–1985 with lower education compared with those with higher education. In China, the education level of children is highly representative of family socioeconomic status and the educational attainment of parents influences the education of their children [31]. Results of this study are similar to a recent report from the United States indicating that trends in AAM vary by socioeconomic position, with lower socioeconomic position associated with higher AAM in the National Health Examination Survey I conducted in 1959–1962 and younger AAM in the National Health Examination and Nutrition Surveys conducted in 2005 to 2008 [44]. Likewise, a study from Brazil showed faster decline in AAM among girls with less-educated fathers than those with better-educated fathers [19].

For urban women and those with higher education, the rate of decline in AAM was smallest in the youngest cohort born 1976–1985 compared with women born 1966–1975. These women may have benefitted from better nutrition *in utero* and better living conditions in childhood and so their AAM was less impacted by the socioeconomic changes occurring during childhood and adolescence. However, we note that the mean AAM consistently remained higher among women in rural regions and those with lower education within each 5-year birth cohort. Similar findings have been reported by previous studies in China showing higher mean AAM in rural women [20,23,25,37,45] and those with lower education [20,36]. Cross-sectional differences in AAM by socioeconomic status have also been reported in a number of other studies examining women from developing countries [18]. Findings from our study suggest that the rate of decline in AAM in China may be slowing and may stabilize for urban and highly educated women in the near future, but continued decline in AMM is expected for rural and less educated women given continued improvement in economic conditions.

A strength of this study is the large population-level sample size of more than 1.1 million women, which facilitates detection of small changes in AAM over time and allows generalization of the results to the southeastern area of China where the study was conducted. As our study population was relatively homogeneous in geography, race, and living habits, selection and confounding biases due to related factors are unlikely. The data were continuously collected over a 13-year period (1993–2005), with consistent methods from women who were born over a 31-year timeframe including the national famine of 1959–60, the Cultural Revolution of 1966–76, and the following 10-year period of extensive economic reform.

Limitations of this study include the use of retrospective data collected from women who were past their menarcheal age, rather than cross-sectional data collected from teenage girls to determine their exact AAM [46]. It has been suggested that recall of AAM becomes less accurate with age [47]; however, a study that compared prospective data with retrospective data collected approximately 30 years after menarche showed high correlation between recalled and true AAM [48]. All women in our sample were under 50 years of age, with the majority under 30 years, and there is low likelihood of recall bias in our study given that the mean (standard deviation) time interval between AAM and age at registration was 10.7 (3.5) years. As AAM increases, marital age and maternal age increase [49], and selection bias may have been introduced by data collection at the marital physical exam or first prenatal visit resulting in higher AAM for older cohorts born 1955–1965 with higher mean age at registration in the PHCSS and lower AAM for women born 1976–1985 with younger mean age at registration; thus, analyses were controlled for age at registration. Another limitation is that data from the PHCSS do not include women who did not marry or become pregnant. Data from the 1990 census of China indicate that in women aged 15 and older, 21% have never been married. About 95% of individuals who never married and are aged 15 and older in China are younger than 30 [50].

## Conclusion

AAM declined in women born in 1955–1985 in southeastern China with the fastest decline in women born in 1966–1975. For the earliest birth cohorts, 1955–1965, AAM declined faster in women from urban compared with rural regions, and a faster decline in AAM was suggested for women with high school or above education compared with primary school or less education. In contrast, for women in the later cohorts born 1976–1985, AAM declined faster among rural compared with urban women and those with lower education compared with higher education. Further studies are warranted to ascertain specific factors that explain these distinct changes, as well as health outcomes associated with earlier AAM in China.

## Additional file

Additional file 1: Table A1. Comparison of mean age at menarche by region and education in each 5-year birth cohort for women born in southeastern China between 1955 and 1985 and included in the Perinatal Health Care Surveillance System.

## Abbreviations

AAM: Age at menarche
NCMIH: National Center of Maternal and Infant Health
PHCSS: Perinatal Health Care Surveillance System
