# Supplementary material for: Secular trends in age at menarche among women born between 1955 and 1985 in Southeastern China
Source: BMC Womens Health. 2014 Dec 12;14:155. doi: 10.1186/s12905-014-0155-0 (PMC4275952; doi:10.1186/s12905-014-0155-0)
Supplement: Additional file 1: Table A1. — Comparison of mean age at menarche by region and education in each 5-year birth cohort for women born in southeastern China between 1955 and 1985 and included in the Perinatal Health Care Surveillance System. [file 12905_2014_155_MOESM1_ESM.doc]

**Additional file 1**

**Secular trends in age at menarche among women born between 1955 and 1985 in southeastern China**

Yanyu Lyu, Lucia Mirea, Junmin Yang, Ruth Warre, Jun Zhang, Shoo K. Lee, Zhu Li

**Contents**

Table A1. Comparison of mean age at menarche by region and education in each 5-year birth cohort for women born in southeastern China between 1955 and 1985 and included in the Perinatal Health Care Surveillance System

**Table A1. Comparison of mean age at menarche by region and education in each 5-year birth cohort for women born in southeastern China between 1955 and 1985 and included in the Perinatal Health Care Surveillance System**

| **Cohort** | **Region** | | |  | **Education Level** | | | |
| --- | --- | --- | --- | --- | --- | --- | --- | --- |
|  | **Urban**  **mean (SD)** | **Rural**  **mean (SD)** | ***P* value^a^** |  | **High school or above**  **mean (SD)** | **Middle school**  **mean (SD)** | **Primary school or less**  **mean (SD)** | **Trend**  ***P* Value^b^** |
| **1955-60** | 15.65 (1.56) | 15.68 (1.50) | <.0001 |  | 15.53 (1.47) | 15.62 (1.40) | 15.74 (1.57) | <.0001 |
| **1961-65** | 15.41 (1.50) | 15.58 (1.40) | <.0001 |  | 15.36 (1.42) | 15.58 (1.35) | 15.62 (1.46) | <.0001 |
| **1966-70** | 14.87 (1.34) | 15.16 (1.31) | <.0001 |  | 14.78 (1.30) | 15.15 (1.27) | 15.27 (1.38) | <.0001 |
| **1971-75** | 14.72 (1.31) | 14.98 (1.24) | <.0001 |  | 14.68 (1.25) | 15.00 (1.22) | 15.09 (1.34) | <.0001 |
| **1976-80** | 14.62 (1.28) | 14.83 (1.22) | <.0001 |  | 14.58 (1.20) | 14.91 (1.21) | 15.00 (1.35) | <.0001 |
| **1981-85** | 14.61 (1.26) | 14.69 (1.19) | <.0001 |  | 14.58 (1.17) | 14.73 (1.20) | 14.73 (1.30) | <.0001 |

^a^*P* value from Student’s t-test

^b^*P* value from ANOVA
